# The effect of dexmedetomidine on vasopressor requirements in patients with septic shock: a subgroup analysis of the Sedation Practice in Intensive Care Evaluation [SPICE III] Trial

**DOI:** 10.1186/s13054-020-03115-x

**Published:** 2020-07-16

**Authors:** Luca Cioccari, Nora Luethi, Michael Bailey, Yahya Shehabi, Belinda Howe, Anna S. Messmer, Helena K. Proimos, Leah Peck, Helen Young, Glenn M. Eastwood, Tobias M. Merz, Jukka Takala, Stephan M. Jakob, Rinaldo Bellomo, for the ANZICS Clinical Trials Group and the SPICE III Investigators, Yahya Shehabi, Yaseen Arabi, Frances Bass, Rinaldo Bellomo, Simon Erickson, Belinda Howe, Suhaini Kadiman, Colin McArthur, Lynnette Murray, Michael Reade, Ian Seppelt, Jukka Takala, Steve A. Webb, Matthew P. Wise, Yahya Shehabi, Belinda Howe, Rinaldo Bellomo, Yaseen M. Arabi, Michael J. Bailey, Frances Bass, Suhaini Kadiman, Colin McArthur, Lynnette Murray, Michael Reade, Ian Seppelt, Jukka Takala, Steve A. Webb, Matthew P. Wise, Michael J. Bailey, Belinda D. Howe, Lynette Murray, Vanessa Singh

**Affiliations:** 1 Department of Intensive Care, Austin Hospital, The University of Melbourne, Melbourne, Australia; 2 Department of Intensive Care Medicine, Inselspital, Bern University Hospital, University of Bern, Bern, Switzerland; 3 Australian and New Zealand Intensive Care Research Centre, School of Public Health and Preventive Medicine, Monash University, Melbourne, Australia; 4 Institute of Social and Preventive Medicine, University of Bern, Bern, Switzerland; 5 Critical Care and Perioperative Services, School of Clinical Sciences, Monash University, Monash Health, Melbourne, Australia; 6 Clinical School of Medicine, University New South Wales, Sydney, Australia; 7 Cardiovascular Intensive Care Unit (CVICU), Auckland City Hospital, Auckland, New Zealand

**Keywords:** Sepsis, Septic shock, Sedation, Hemodynamics, Dexmedetomidine, Noradrenaline

## Abstract

**Background:**

Septic shock is associated with decreased vasopressor responsiveness. Experimental data suggest that central alpha2-agonists like dexmedetomidine (DEX) increase vasopressor responsiveness and reduce catecholamine requirements in septic shock. However, DEX may also cause hypotension and bradycardia. Thus, it remains unclear whether DEX is hemodynamically safe or helpful in this setting.

**Methods:**

In this post hoc subgroup analysis of the Sedation Practice in Intensive Care Evaluation (SPICE III) trial, an international randomized trial comparing early sedation with dexmedetomidine to usual care in critically patients receiving mechanical ventilation, we studied patients with septic shock admitted to two tertiary ICUs in Australia and Switzerland. The primary outcome was vasopressor requirements in the first 48 h after randomization, expressed as noradrenaline equivalent dose (NEq [μg/kg/min] = noradrenaline + adrenaline + vasopressin/0.4).

**Results:**

Between November 2013 and February 2018, 417 patients were recruited into the SPICE III trial at both sites. Eighty-three patients with septic shock were included in this subgroup analysis. Of these, 44 (53%) received DEX and 39 (47%) usual care. Vasopressor requirements in the first 48 h were similar between the two groups. Median NEq dose was 0.03 [0.01, 0.07] μg/kg/min in the DEX group and 0.04 [0.01, 0.16] μg/kg/min in the usual care group (*p* = 0.17). However, patients in the DEX group had a lower NEq/MAP ratio, indicating lower vasopressor requirements to maintain the target MAP. Moreover, on adjusted multivariable analysis, higher dexmedetomidine dose was associated with a lower NEq/MAP ratio.

**Conclusions:**

In critically ill patients with septic shock, patients in the DEX group received similar vasopressor doses in the first 48 h compared to the usual care group. On multivariable adjusted analysis, dexmedetomidine appeared to be associated with lower vasopressor requirements to maintain the target MAP.

**Trial registration:**

The SPICE III trial was registered at ClinicalTrials.gov (NCT01728558).

## Background

In septic shock, sympathetic activation and release of endogenous catecholamines are necessary physiological mechanisms to maintain adequate tissue perfusion [1]. However, excessive release of endogenous catecholamines in combination with exogenous catecholamines can cause sympathetic overstimulation with detrimental effects on organ function and patient outcomes [2]. Sympathetic overstimulation may also cause downregulation and desensitization of alpha-adrenergic receptors. Such catecholamine hyposensitivity may also contribute to hemodynamic instability and poor outcomes [3, 4].

In experimental animal models of sepsis, high doses of central alpha-2-agonists like clonidine and dexmedetomidine increase vasopressor responsiveness [5]. Moreover, even in non-septic patients, alpha-2-agonists are associated with lower vasopressor requirements [6, 7], increased arterial blood pressure, and enhanced baroreceptor response [8, 9]. However, significant hemodynamic side effects of dexmedetomidine can also occur such as hypotension and bradycardia [10].

In the Sedation Practice in Intensive Care Evaluation (SPICE III) Trial, critically ill patients on mechanical ventilation received early dexmedetomidine for primary sedation or usual sedation [11]. The study design and randomization scheme provided the unique opportunity to explore the hemodynamic effects of dexmedetomidine in patients with septic shock. Thus, in this two-center retrospective subgroup analysis of patients with septic shock included in the SPICE III trial, we assessed the physiological effects of dexmedetomidine on vasopressor requirements and blood pressure in the first 48 h after randomization.

## Methods

The SPICE III trial was a randomized, open-label trial conducted at 74 sites in eight countries [11]. The study complied with the Declaration of Helsinki and Good Clinical Practices and was approved by the institutional review board at participating centers. Written informed consent was obtained for all patients. The study design, protocol, and statistical analysis plan have been previously published [12]. Critically ill adults (18 years or older) receiving mechanical ventilation for less than 12 h in the intensive care unit (ICU) were randomized to receive dexmedetomidine as the sole or primary sedative or to receive usual care (propofol, midazolam, or other sedatives), if they were expected to remain on invasive ventilatory support for longer than the next calendar day. The primary outcome of the SPICE III trial was the rate of death from any cause at 90 days.

### Study design

The present study is an exploratory, post hoc, retrospective subgroup analysis of patients with septic shock who were enrolled in the SPICE trial III. The subgroup analysis was performed at two of the participating centers, the Austin Hospital, Melbourne, Australia, and the University Hospital of Bern, Switzerland, and was approved by the ethics committees at both sites (approval number LNR/15/Austin/391 and KEK-ID2018-00746).

### Inclusion and exclusion criteria

In addition to the abovementioned inclusion criteria of the SPICE III trial, patients had to meet all the following criteria to be eligible for this subgroup analysis: documented (or strong suspicion of) infection with at least 2 clinical signs of inflammation (temperature > 38 °C or < 36 °C, heart rate > 90/min, respiratory rate > 20/min, or PaCO_2_ < 32 mmHg, white cell count > 12 × 10^9^/l or < 4 × 10^9^/l or > 10% immature neutrophils), and administration of vasopressors or inotropes prior to randomization and for a cumulative duration of ≥ 4 h to maintain blood pressure targets set by the treating clinician.

In addition to the exclusion criteria of the SPICE III trial, we excluded patients meeting our inclusion criteria more than 24 h before randomization. A detailed list of all inclusion and exclusion criteria is given in the supplementary appendix. To reflect the effects of the intervention (dexmedetomidine or usual care) unaffected by protocol deviations or non-adherence, we performed a per-protocol analysis excluding patients who did not receive the allocated sedation regimen and patients whose treatment goal was changed to end-of-life care within the first 48 h after randomization.

### Clinical outcomes

The primary outcome was the median vasopressor dose in the first 48 h after randomization, expressed as noradrenaline equivalent dose (NEq). We calculated NEq using the method described by Khanna and co-workers [13] as

$$ \mathrm{NEq}\ \left[\upmu \mathrm{g}/\mathrm{kg}/\min \right]=\mathrm{noradrenaline}+\mathrm{adrenaline}+\mathrm{vasopressin}/0.4 $$

to account for the different vasopressors used at the two study sites. Secondary outcomes included cumulative and peak NEq dose, change in NEq dose from baseline to peak dose, and the ratio of NEq to MAP (NEq/MAP), to account for different blood pressure targets set by the treating physician. Exploratory outcomes included cumulative duration of vasopressor support, ICU and hospital mortality, duration of mechanical ventilation, and ICU length of stay.

### Data collection

Data collection was performed using existing ICU-based electronic databases (Cerner Electronic Health Record, North Kansas City, MO, USA, and Centricity Critical Care, Clinisoft, GE Healthcare Europe, Helsinki, Finland) and medical record review. Whenever possible, data were downloaded from electronic medical records. Continuous measurements, recorded at minimum once every 2 min, were resampled to obtain hourly median values of hemodynamic data, sedative and vasoactive drug doses for the first 48 h after randomization. Arterial blood pressure was monitored invasively in all patients. Where manual data collection was necessary, it was performed using double data entry or cross-checked by a second investigator. Demographic data and patient-centered outcomes were obtained from the SPICE III database.

### Statistical analysis

Data was initially assessed for normality and log-transformed where appropriate. Group comparisons were performed using chi-square tests for equal proportion, Student’s *t* tests for normally distributed data, and Wilcoxon rank-sum tests otherwise, with results presented as numbers (%), means (standard deviations), or medians (interquartile range), respectively. Longitudinal analysis was performed using mixed linear modeling fitting main effects for treatment, time, and an interaction between the two to determine if groups behaved differently over time. Multivariable longitudinal sensitivity analyses were performed firstly adjusting for baseline imbalance and known covariates (admission diagnosis, hospital site, ratio of noradrenaline equivalent over mean arterial pressure at baseline, continuous renal replacement therapy, age, administration of hydrocortisone, and presence of liver cirrhosis) and then secondly adjusting for the same covariates but with treatment group replaced by actual dexmedetomidine dosage. Comparison of proportions for secondary outcomes was determined using logistic regression with results presented as odds ratios (95%CI). Differences for continuous secondary outcomes were determined using quantile regression with results presented as difference of medians (95%CI). To account for the competing risk of death, durations of vasopressor support and ventilation are presented as cumulative incidence functions with censoring for death and comparison using Grey’s test. Time to death was displayed using Kaplan-Meier curves with comparison using a log-rank test. All data were analyzed using SAS software, version 9.4 (SAS Institute Inc., Cary, NC, USA), and a two-sided *p* value of 0.05 was used to indicate statistical significance. No adjustments were made for multiple comparisons.

### Sample size

With a minimum of 38 patients per group, this study had > 90% power (2-sided *p* value) to detect a difference in noradrenaline requirement equivalent to 75% of one standard deviation. Across the range of the data, a difference of this magnitude approximately equates to a 20% difference, which is perceived to be of clinical importance.

## Results

### Characteristics of the patients

Between November 2013 and February 2018, 417 patients were recruited in the SPICE III trial at both sites (196 patients at the Austin Hospital and 221 patients at the University Hospital of Bern). Among those who provided written informed consent, 87 patients fulfilled our inclusion criteria of septic shock. After excluding two patients from each group who did not receive the assigned sedation, 83 patients were included in the analysis. Of these, 44 (53%) had been assigned to receive dexmedetomidine (DEX group) and 39 (47%) to usual care. Fifty-seven patients (68.7%) were male, with a mean age of 65.4 years, and a mean pre-randomization APACHE II score of 25.1. Patient characteristics and treatment at randomization were similar in the two groups, except for a higher creatinine level and higher coagulation component of the SOFA score in the usual care group (Table 1).

**Table 1:** Patient characteristics at baseline

Variable	DEX (***n*** = 44)	Usual care (***n*** = 39)	***p*** value
Study site—no. (%)			0.77
Australia	20 (45.5)	19 (48.7)	
Switzerland	24 (54.5)	20 (51.3)	
Age (years)—mean ± SD	67.7 ± 12.4	62.9 ± 16.8	0.14
Male sex—no. (%)	29 (65.9)	28 (71.8)	0.56
Weight (kg)—mean ± SD	80.6 ± 17.7	85.3 ± 31.4	0.39
APACHE II score—mean ± SD	24.9 ± 6.7	25.3 ± 7.0	0.77
Chronic health conditions—no.(%)			
Diabetes mellitus treated with insulin	4 (9.1)	2 (5.1)	0.68
Chronic hemodialysis	1 (2.3)	1 (2.6)	> 0.99
Liver cirrhosis	1 (2.3)	1 (2.6)	> 0.99
Portal hypertension	1 (2.3)	3 (7.7)	0.34
Immunosuppression by disease	1 (2.3)	2 (5.1)	0.60
Immunosuppression by therapy	2 (4.5)	3 (7.7)	0.66
ICU admission source—no. (%)			0.91
Emergency department	13 (29.5)	13 (33.3)	
Hospital ward	18 (40.9)	13 (33.3)	
Operating room	7 (15.9)	9 (23.1)	
Another ICU	1 (2.6)	1 (2.3)	
Other hospitals	5 (11.4)	3 (7.7)	
Surgical admission—no. (%)	8 (18.2)	10 (25.6)	0.41
Primary site of infection—no. (%)			
Respiratory	26 (59.1)	18 (46.2)	0.24
Gastrointestinal	10 (22.7)	14 (35.9)	0.19
Skin/soft tissues/bone	3 (6.8)	4 (10.3)	0.7
Urinary	1 (2.3)	1 (2.6)	> 0.99
Blood	2 (4.5)	0 (0.0)	0.5
Other	2. (4.5)	2 (5.1)	> 0.99
Organ-specific SOFA score—median [IQR]			
Cardiovascular	3 [3, 3]	3 [3, 4]	0.06
Respiratory	2 [2, 3]	2 [2, 3]	0.80
Renal	1 [0, 3]	2 [0, 3]	0.27
Coagulation	0 [0, 0]	1 [0, 2]	0.006
Liver	0 [0, 1]	1 [0, 2]	0.14
NEq (μg/kg/min)—median [IQR]	0.05 [0.03, 0.10]	0.07 [0.02, 0.16]	0.32
Continuous vasoactive drugs at baseline—no. (%)			
Noradrenaline	38 (86.4)	35 (89.7)	0.64
Adrenaline	4 (9.1)	2 (5.1)	0.68
Dobutamine	1 (2.3)	3 (7.7)	0.34
Vasopressin	1 (2.3)	2 (5.1)	0.60
Sedative and analgesic drugs at baseline—no. (%)			
Propofol	31 (73.8)	33 (86.8)	0.15
Fentanyl	26 (61.9)	30 (78.9)	0.10
Midazolam	20 (47.6)	16 (42.1)	0.62
Morphine	5 (11.9)	3 (7.9)	0.55
Ketamine	2 (4.8)	2 (5.3)	> 0.99
Other treatments at baseline—no. (%)			
Continuous renal replacement therapy	10 (22.7)	13 (33.3)	0.28
Hydrocortisone^a^ for septic shock	19 (43.2)	16 (41.0)	0.84
Physiological variables			
Fluid balance at baseline (ml)—median [IQR]	876 [− 21, 2600]	621 [− 67, 2378]	0.88
Heart rate (beats/min)—median [IQR]	85 [74, 99.5]	95 [80, 105]	0.10
Mean arterial pressure (mmHg)—mean ± SD	65.4 ± 8.35	66.1 ± 8.85	0.71
Creatinine level (mg/dl)—median [IQR]	1.23 [0.78, 2.12]	1.76 [1.14, 2.34]	0.044
Creatinine level (μmol/l)—median [IQR]	109 [69, 187]	156 [101, 207]	0.044
Lactate level (mmol/l)—median [IQR]	1.8 [1.4, 2.7]	1.95 [1.4, 3.1]	0.58
RASS prior randomization—median [IQR]	− 3 [− 4, 1]	− 3 [− 4, − 2]	0.69
Time from ICU admission to randomization (h)—median [IQR]	8.8 [3.6, 12.4]	11.1 [4.7, 19.1]	0.22
Time from ICU admission to start of vasopressors (h)—median [IQR]	1.4 [0.5, 3.5]	2.7 [0.4, 5.1]	0.39

Categorical values are expressed as numbers (%). Continuous variables are presented as means ± SD if normally distributed, otherwise as medians [IQR]

*APACHE* Acute Physiology And Chronic Health Evaluation, *DEX* dexmedetomidine, *ICU* intensive care unit, *NEq* noradrenaline equivalents, RASS: Richmond Agitation-Sedation Scale, *SOFA* Sequential Organ Failure Assessment

^a^In the first 48 h after randomization

### Clinical outcomes

**Fig. 1:**
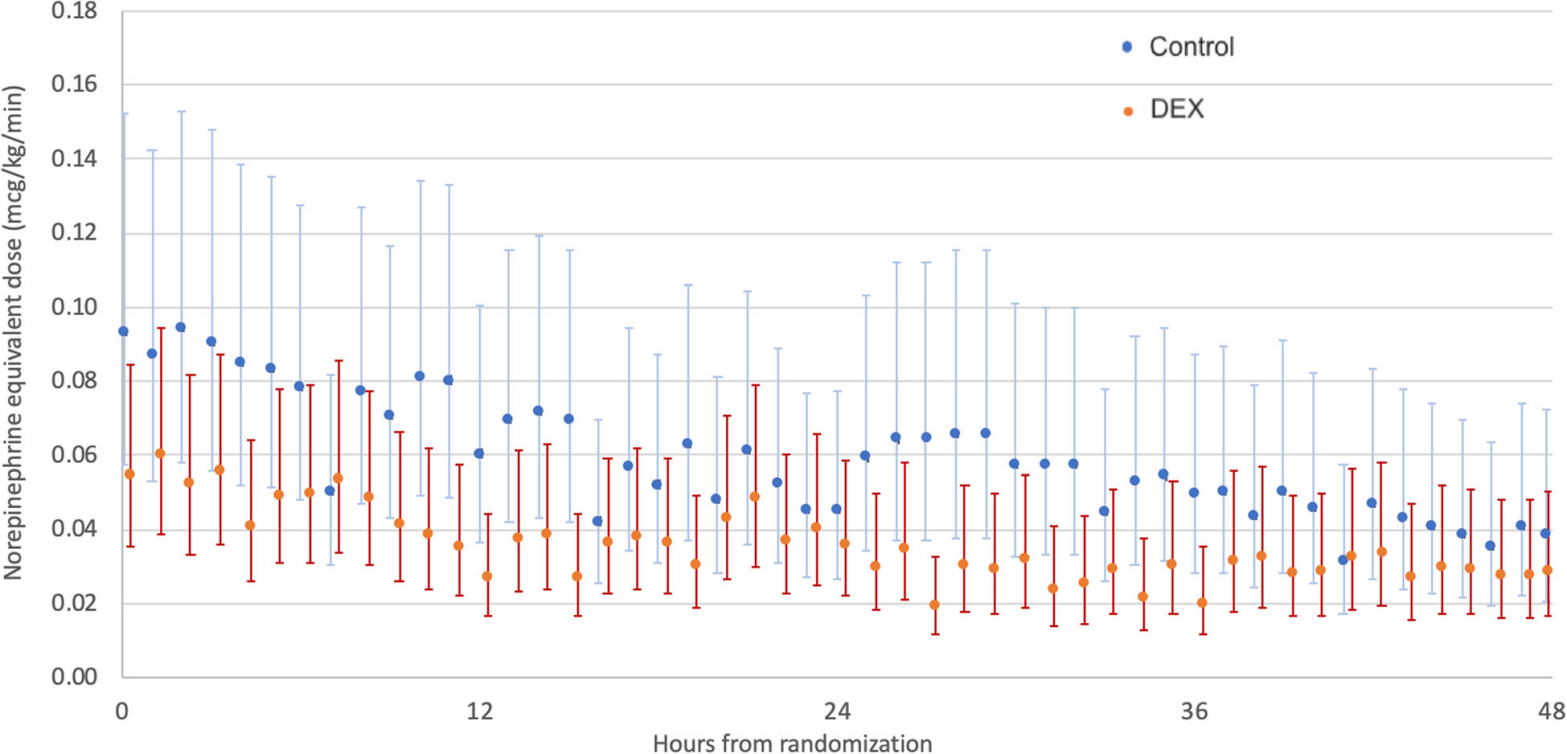
Noradrenaline equivalent dose in the first 48 h after randomization. Data are presented as geometric means and 95% confidence intervals, overall group difference *p* = 0.054

In the first 48 h after randomization, there was no significant difference in median NEq dose between the DEX group and the usual care group (Table 2 and Fig. 1). Similarly, we found no significant difference in cumulative NEq dose, peak NEq dose, and relative change in NEq from baseline to peak dose between the two groups.

**Table 2:** Clinical outcomes

Outcome	DEX (***n*** = 44)	Usual care (***n*** = 39)	Estimate (95%CI)	***p*** value
**Difference in medians**				
NEq dose^a^, μg/kg/min	0.03 [0.01, 0.07]	0.04 [0.01, 0.16]	− 0.01 [− 0.06, 0.04]	0.17
Noradrenaline dose^a^, μg/kg/min	0.03 [0.01, 0.07]	0.05 [0.01, 0.15]	− 0.01 [− 0.06, 0.03]	0.08
Cumulative NEq dose^a^, μg/kg/48 h.	1.51 [0.51, 3.60]	2.14 [0.58, 7.78]	− 0.62 [− 3.18, 1.93]	0.19
Peak NEq dose^a^, μg/kg/min	0.12 [0.05, 0.20]	0.16 [0.08, 0.32]	− 0.03 [− 0.12, 0.06]	0.24
Change in NEq dose from baseline to peak level^a^, μg/kg/min	0.05 [0.01, 0.12]	0.05 [0.01, 0.14]	− 0.01 [− 0.06, 0.05]	0.61
Total duration of vasopressor support^b^, h	51.6 [18.3, 99.7]	45.7 [19.6, 159.0]	3.1 [− 30.7, 36.9]	0.72
Survivors (*n* = 67)	35.6 [18.3, 69.4]	40.3 [22.2, 75.6]	− 3.5 [− 30.1, 23.1]	0.64
Non-survivors (*n* = 16)	186.0 [59.0, 311.0]	70.4 [19.4, 168.0]	19.8 [− 173.1, 212.6]	0.42
Duration of invasive ventilation^b^, days	2.2 [1.1, 5.9]	2.8 [1.2, 9.6]	− 0.5 [− 3.8, 2.8]	0.60
Hospital length of stay^b^, days	15.5 [9.4, 24.4]	13.2 [7.7, 21.0]	− 2.2 [− 8.5, 4.1]	0.30
ICU length of stay^b^, days	4.2 [2.7, 10.2]	4.7 [3.0, 11.3]	− 0.4 [− 3.4, 2.6]	0.67
Survivors (*n* = 67)	4.1 [3.0, 9.2]	4.3 [3.2, 7.0]	− 0.1 [− 2.7, 2.3]	0.70
Non-survivors (*n* = 16)	8.9 [2.4, 16.2]	8.3 [0.8, 11.3]	− 3.3 [− 16.2, 9.6]	0.87
**Odds ratio (95% CI)**				
Patients alive and vasopressor-free at 48 h after randomization	20 (45.5)	18 (46.2)	0.97 (0.41–2.31)	0.95
ICU mortality	6 (13.6)	10 (25.6)	0.46 (0.15–1.41)	0.17
Hospital mortality	9 (20.5)	12 (30.8)	0.58 (0.21–1.57)	0.28
Day 90 mortality	12 (27.3)	13 (34.2)	0.72 (0.28–1.85)	0.50

Categorical values are expressed as numbers (%). Continuous variables are presented as medians [IQR]

*DEX* dexmedetomidine, *IQR* interquartile range, *95% CI* 95% confidence interval, *ICU* intensive care unit, *MAP* mean arterial pressure, *NEq* noradrenaline equivalents

^a^In the first 48 h after randomization

^b^Within principal hospital admission, measured from randomization

**Fig. 2:**
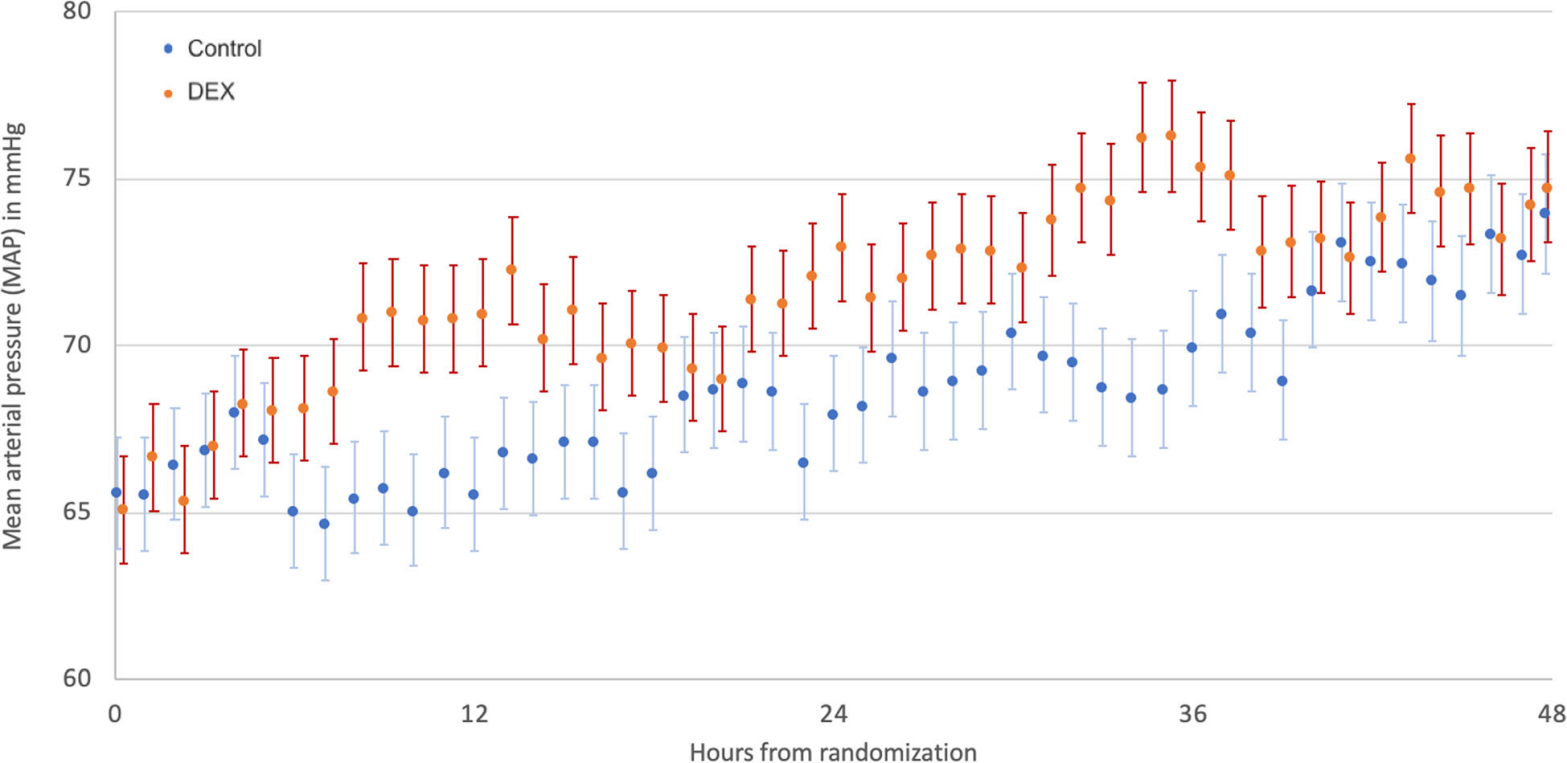
Mean arterial pressure in the first 48 h after randomization. Data are presented as mean with standard error, overall group difference *p* = 0.06

**Fig. 3:**
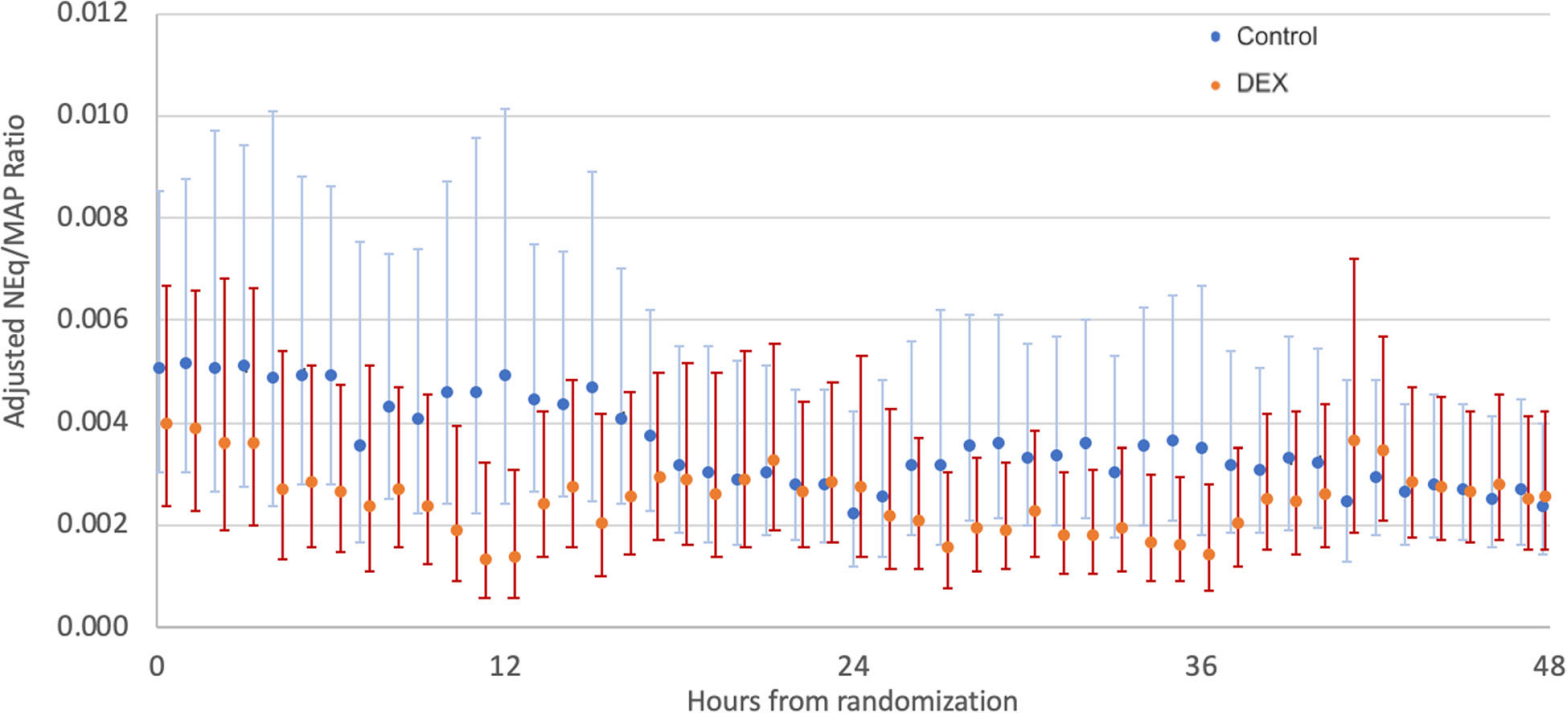
Adjusted ratio of noradrenaline equivalents divided by MAP (NEq/MAP ratio) in the first 48 h after randomization. Adjusted for admission diagnosis, hospital site, baseline NEq/MAP ratio, continuous renal replacement therapy, age, administration of hydrocortisone and presence of liver cirrhosis. NEq/MAP: Noradrenaline equivalents to mean arterial pressure ratio (a higher ratio indicates higher vasopressor need to maintain a certain MAP). Data are presented as geometric means and 95% confidence intervals, overall group difference *p* = 0.02

Over the first 48 h, patients in the DEX group had a numerically higher MAP (Fig. 2), although the differences did not reach statistical significance. However, vasopressor requirements to maintain the target MAP (expressed by the NEq/MAP ratio) were lower in the DEX group compared to the usual care group (ratio of difference in geometric means 1.74 [1.02, 2.95], *p* = 0.04). This result remained significant when adjusted for admission diagnosis, hospital site, baseline NEq/MAP ratio, continuous renal replacement therapy, age, administration of hydrocortisone, and liver cirrhosis (ratio of adjusted difference in geometric means 1.44 [1.07–1.95], *p* = 0.02) (Fig. 3).

On multivariable sensitivity analysis adjusting for actual dexmedetomidine dosage, the results were consistent with the above findings: Dexmedetomidine dose was associated with a reduction in the NEq/MAP ratio (parameter estimate of − 0.17 +/− 0.07; *p* = 0.02), indicating lower vasopressor requirements to maintain the target MAP. The NEq/MAP ratio was significantly greater in patients treated in Australia, in patients not on continuous renal replacement therapy, without liver disease, without portal hypertension, or with a renal or cardiovascular APACHE admission category (Table 3).

**Table 3:** Multivariable adjusted analysis of the association between hourly dexmedetomidine dose and noradrenaline equivalent to MAP ratio (NEq/MAP)

Effect	Estimate	Standard error	***p*** value
Dexmedetomidine, per μg/kg/h increase	− 0.165	0.071	0.02
Age, per year increase	− 0.003	0.003	0.38
Baseline log NEq/MAP, per unit increase	0.312	0.059	< 0.001
Location			
Switzerland	− 0.672	0.101	< 0.001
Australia	0		
No hydrocortisone	0.134	0.108	0.22
hydrocortisone	0		
No CRRT	− 0.635	0.105	< 0.001
CRRT	0		
No liver cirrhosis	− 1.371	0.247	< 0.001
Liver cirrhosis	0		
No portal hypertension	− 0.477	0.174	0.008
Portal hypertension	0		
APACHE III admission diagnosis			
Cardiovascular	0.678	0.272	0.02
Gastrointestinal	0.186	0.125	0.14
Hematological	0.091	0.696	0.90
Musculoskeletal	− 0.286	0.365	0.44
Renal	2.399	0.317	< 0.001
Respiratory	0.086	0.111	0.44
Sepsis	0		

Adjusted for admission diagnosis, hospital site, baseline NEq/MAP ratio, continuous renal replacement therapy, age, administration of hydrocortisone, and presence of liver cirrhosis

*APACHE* Acute Physiology And Chronic Health Evaluation, *CRRT* continuous renal replacement therapy, *MAP* mean arterial pressure, *NEq* noradrenaline equivalents, *NEq/MAP* noradrenaline equivalents to MAP ratio (a higher ratio indicates higher vasopressor need to maintain a certain MAP)

**Fig. 4:**
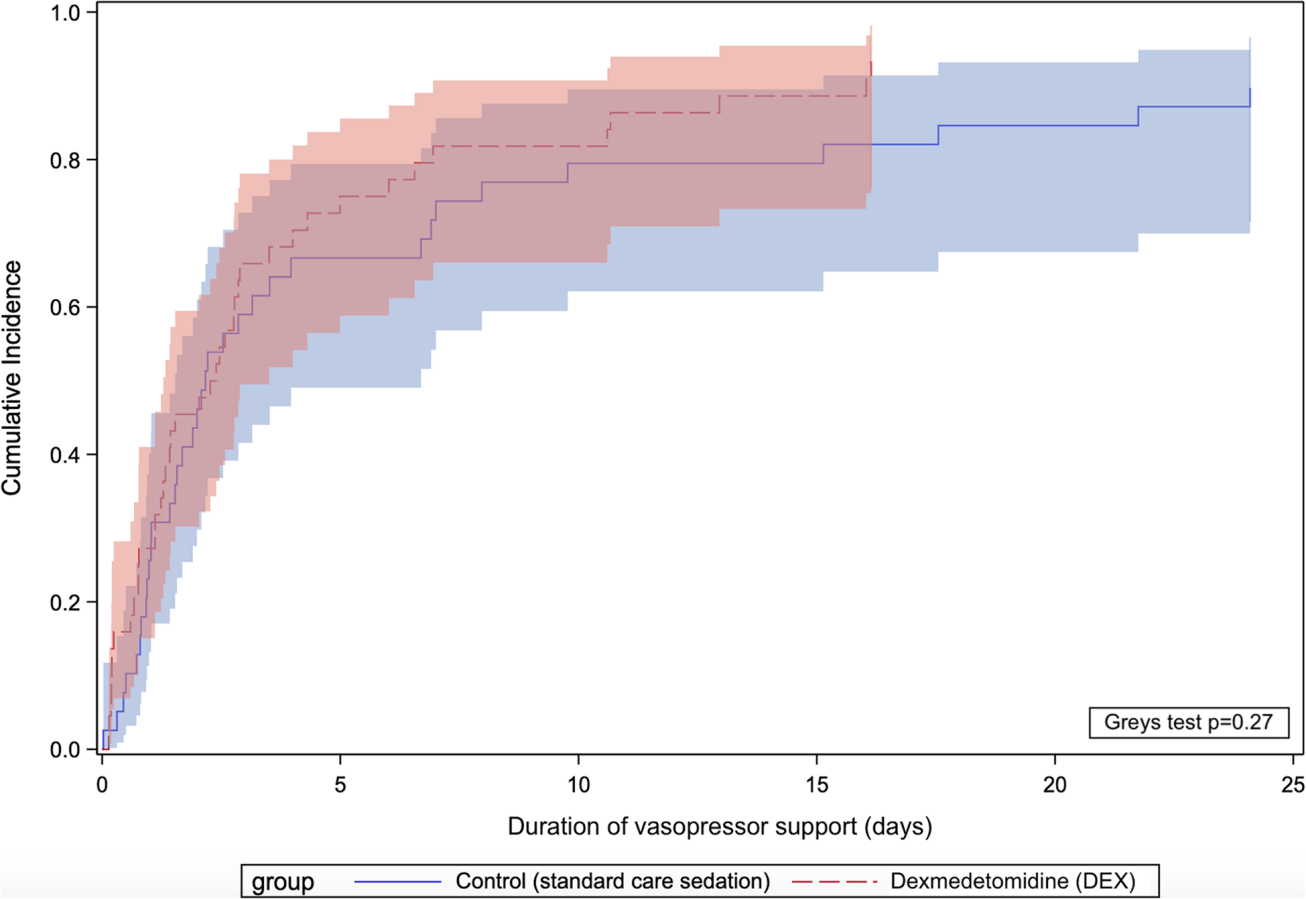
Cumulative incidence curves for time to vasopressor weaning (with deaths treated as a competing risk) and comparison using Grey’s test. Shaded areas represent 95% confidence intervals

**Fig. 5:**
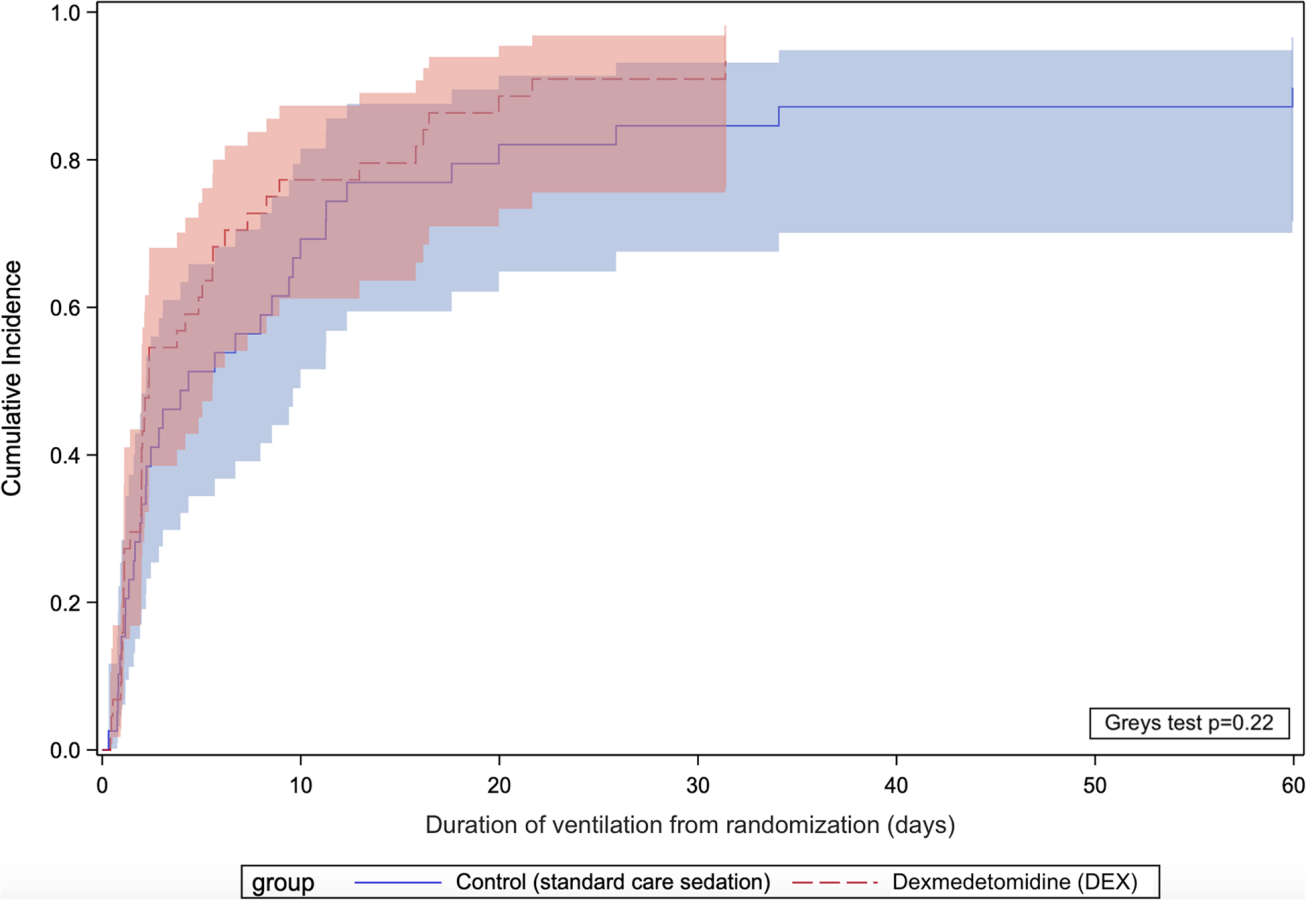
Cumulative incidence curves for the duration of invasive ventilation from randomization (with deaths treated as a competing risk) and comparison using Grey’s test. Shaded areas represent 95% confidence intervals

**Fig. 6:**
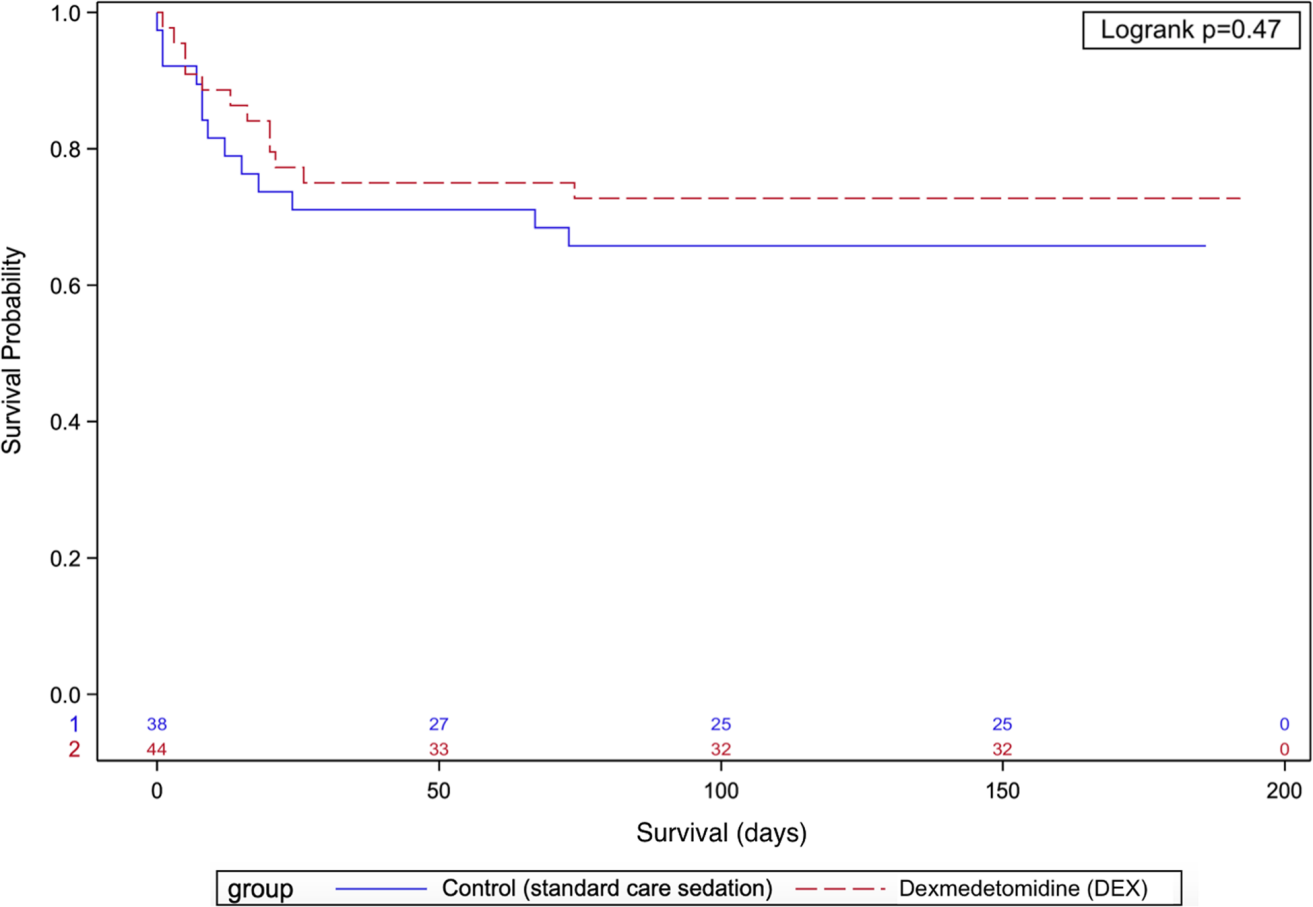
Kaplan-Meier curve for the probability of survival, with the number of subjects at risk

There was no significant difference in ICU and hospital length of stay, number of patients alive and vasopressor-free at 48 h, time to vasopressor weaning, duration of invasive ventilation, and mortality between the two groups (Table 2 and Figs. 4, 5, and 6). Unlike the SPICE III trial, in the present sub-study, we found no significant difference in outcomes when comparing age groups above and below the median age (63.7 years) (Table S4).

### Process of care

Nineteen patients (43.2%) in the DEX group and 16 patients (41.0%) in the usual care group received hydrocortisone (*p* = 0.84). Vasoactive drugs were discontinued within 48 h of randomization in 21 (47.7%) patients in the DEX group and 21 (53.8%) patients in the usual care group (*p* = 0.58). Patients in the DEX group received significantly lower doses of propofol and less midazolam. However, they received similar doses of opioids (Table S1). The median Richmond Agitation-Sedation Scale (RASS) score on study day 1 was − 4 [− 4, − 2] in the DEX group and − 4 [− 4, − 3] in the usual care group (*p* = 0.42). There was no significant difference regarding treatment with vasopressors, inotropes, and hydrocortisone between patients treated before and after the publication of the 2016 sepsis guidelines (Table S5).

### Adverse events

Adverse events were prospectively collected during the original SPICE III trial. Due to the un-blinded study design, events were reported by site investigators but not systematically collected in both groups. Bradycardia was defined as heart rate < 55 beats per minute requiring intervention (e.g., pacing, pharmacological support, or modification of dexmedetomidine or other medication doses). Hypotension was defined as hypotension which is clinically significant in the Principal Investigator’s opinion. In our cohort, hypotension was reported in seven (15.9%) patients in the DEX group and one (2.6%) patient in the usual care group (*p* = 0.04). Bradycardia occurred in five patients (11.4%), all were from the DEX group (*p* = 0.06). Serious adverse events occurred in two patients in the DEX group (hypotension) and one patient in the usual care group (cardiac arrest, survived) (Table S2).

## Discussion

In this exploratory post hoc retrospective subgroup analysis of ICU patients with septic shock requiring mechanical ventilation, patients receiving early sedation with dexmedetomidine as the primary sedative agent had similar vasopressor requirements in the first 48 h compared to usual care. On adjusted exploratory analysis, dexmedetomidine appeared to be associated with lower vasopressor requirements to maintain the target MAP.

As alpha-2 agonist, dexmedetomidine may increase the risk of hypotension and bradycardia [14, 15] and, in patients with septic shock, this may be both dangerous and harmful. However, experimental data also suggest that dexmedetomidine can be administered in septic shock and may even have catecholamine-sparing effects [5, 16–21]. Our findings are consistent with such experimental observations. In a subgroup analysis of septic patients (*n* = 63) from the Maximizing Efficacy of Targeted Sedation and Reducing Neurological Dysfunction (MENDS) trial, the incidence of hypotension, vasopressor use, and cardiac arrhythmias were similar in both groups (dexmedetomidine vs. lorazepam-based sedation), but the trial was not powered to detect a difference in cardiovascular outcomes [22]. A retrospective study by Nelson et al. compared clinically significant hemodynamic events in adults with septic shock receiving dexmedetomidine (*n* = 37) or propofol (*n* = 35) (Nelson 2018). Dexmedetomidine was not associated with more episodes of hypotension or bradycardia (29.7% vs. 31.4, *p* = 0.99) [23]. A prospective open-label crossover study in 38 patients with septic shock found a reduction of catecholamine requirements after switching from propofol to dexmedetomidine [18]. However, both studies included resuscitated patients on stable vasopressor doses for at least 2 h, and, in the latter study, data collection was limited to the first 8 h.

In animal models of sepsis, high doses of central alpha2-agonists increase vasopressor responsiveness [5]. Moreover, dexmedetomidine reduces noradrenaline requirements and maintains renal function in experimental septic acute kidney injury [17]. In healthy volunteers, dexmedetomidine decreases plasma levels of both noradrenaline and adrenaline [9]. The mechanisms behind these findings are not fully understood, and different hypotheses have been proposed to explain how alpha-2 agonists improve vasopressor responsiveness. One hypothesis is that alpha-2 agonists have the potential to prevent downregulation and/or lead to resensitization of alpha-1 adrenergic receptors by reducing the sympathetic outflow and the release of endogenous catecholamines in sepsis [4, 24]. Presumably, lowering endogenous noradrenaline levels improves the action of exogenous noradrenaline on vascular α-1 receptors [5]. The second hypothesis suggests an anti-inflammatory effect of DEX [25–30], mediated by the vagus nerve and the so-called cholinergic anti-inflammatory pathway [31], a neural circuit that responds to and regulates the inflammatory response via the release of acetylcholine [8]. Finally, α-2 agonists may act via local vascular mechanisms on vascular smooth muscle cells [32] and the presynaptic action of α-2 agonists could, by reducing the release of endogenous noradrenaline, lead to an upregulation of postsynaptic α-1 receptors [5].

Our findings that sedation with dexmedetomidine does not increase vasopressor requirements in the first 48 h imply that dexmedetomidine may be used in patients with septic shock without worsening hemodynamic instability. Our findings neither support nor refute the hypothesis that dexmedetomidine has a catecholamine-sparing effect in this setting, although the association of dexmedetomidine with a lower NEq/MAP ratio points towards lower overall vasopressor requirements to maintain the target MAP. However, because we did not pre-specify this outcome and we did not adjust for multiple comparisons, this finding should be considered exploratory. Moreover, the percentage of RASS scores below − 2 on study day 1 was higher in the usual care group compared to the DEX group, indicating deeper sedation in the usual care group in the first 24 h. This may, at least in part, explain the lower vasopressor requirements in the DEX group. Notwithstanding these limitations, the observed trend towards higher MAP in the DEX group and its potential to lower vasopressor requirements support further explorations of dexmedetomidine-based sedation in mechanically ventilated patients with septic shock.

### Strengths and limitations

Our study has several strengths. First, we studied patients in septic shock randomly assigned to one of two treatment strategies (early DEX sedation vs. usual care) and analyzed the hemodynamic effects of dexmedetomidine compared to usual care sedation. Second, we obtained granular and detailed data from two academic ICUs on hemodynamic parameters, level of sedation, dose, and duration of vasoactive and sedative drugs. Third, we used data from the original SPICE III database, electronic patient health records (whenever possible), and double data entry for manually collected data, to minimize the risk of ascertainment bias.

Our study is a retrospective exploratory subgroup analysis, not prespecified in the SPICE III study protocol and, therefore, comes with inevitable limitations. All findings must be interpreted strictly as hypothesis generating. However, due to the existing clinical equipoise regarding benefits and risks of using dexmedetomidine in patients with septic shock, our results have incremental value in adding to our understanding of the hemodynamic effects of dexmedetomidine in such patients. Because the goal of this investigation was to explore the effects of dexmedetomidine on hemodynamic parameters and vasopressor use, we excluded two patients in each group who did not receive the assigned treatment and opted for a per-protocol instead of an intention-to-treat analysis. However, baseline characteristics of patients included were similar, and it is unlikely that including these four patients would have materially altered our results. Hemodynamic management was not dictated by protocol and, as management of vasodilatory shock differs with respect to utilization of steroids and vasopressors [33], we cannot exclude a bias related to the open-label design of the SPICE III study or due to clinical preferences. However, by calculating the NEq/MAP ratio we sought to account for the different types of vasopressors used and for the individual MAP targets prescribed by the treating clinicians; the proportion of patients treated with hydrocortisone was similar in both groups, and we included hydrocortisone in our multivariable adjusted analysis. Finally, DEX was associated with more episodes of bradycardia and hypotension, as already reported in the SPICE trial (11). Thus, although the overall effect of using DEX as the primary sedating agent in mechanically ventilated patients with septic shock appears to improve the vasopressor dose to MAP ratio in the first 48 h, in some patients it may precipitate unwanted hemodynamic changes. Accordingly, caution should be exercised when starting the infusion and in administering this agent in patients at risk of bradycardia.

## Conclusion

In critically ill patients with septic shock, compared to usual care, patients receiving early sedation with dexmedetomidine as the primary sedative agent had similar vasopressor requirements in the first 48 h compared to usual care. On multivariable adjusted analysis, dexmedetomidine appeared to be associated with lower vasopressor requirements to maintain the target MAP. These findings should be interpreted as exploratory and hypothesis generating. However, they provide a rationale for further randomized trials evaluating dexmedetomidine in patients with septic shock.

## Supplementary information

**Additional file 1.**

**Additional file 2.**

## Data Availability

The data generated and/or analyzed during the current study are part of the SPICE III trial and, therefore, not publicly available. De-identified data that underly the reported results will be made available to researchers with a defined protocol and analysis plan, after approval by Monash University, Melbourne, the University of Bern, Switzerland, and the SPICE III Management Committee (Email to yahya.shehabi@monash.edu or y.shehabi@unsw.edu.au).

## Abbreviations

APACHE: Acute Physiology And Chronic Health Evaluation
CI: Confidence interval
DEX: Dexmedetomidine
ICU: Intensive care unit
IQR: Interquartile range
MAP: Mean arterial pressure
NEq: Noradrenaline equivalents
NEq/MAP: Noradrenaline equivalents to MAP ratio
RASS: Richmond Agitation-Sedation Scale
RRT: Renal replacement therapy
SOFA: Sequential Organ Failure Assessment
SPICE: Sedation Practice in Intensive Care Evaluation
