# Supplementary material for: The effect of dexmedetomidine on vasopressor requirements in patients with septic shock: a subgroup analysis of the Sedation Practice in Intensive Care Evaluation [SPICE III] Trial
Source: Crit Care. 2020 Jul 16;24:441. doi: 10.1186/s13054-020-03115-x (PMC7367420; doi:10.1186/s13054-020-03115-x)
Supplement: Supplementary file 1 — Additional file 1. [file 13054_2020_3115_MOESM1_ESM.docx]

**Additional file 1**

Supplement to: Cioccari L, Luethi N, Bailey M, et al. The effect of dexmedetomidine on vasopressor requirements in patients with septic shock: a subgroup analysis of the Sedation Practice in Intensive Care Evaluation [SPICE III] Trial.

**Appendix**

**1. Inclusion and exclusion criteria**

**Inclusion criteria (all criteria must be met):**

1. Subject has been intubated and is receiving mechanical ventilation.
2. The treating clinician expects that the patient will remain intubated until the day after

tomorrow (unlikely to be extubated the following day).

1. The patient requires immediate ongoing sedative medication for comfort, safety, and to facilitate the delivery of life support measures.
2. Documented site, or strong suspicion of infection with two of the 4 clinical signs of inflammation (within the prior 24 hours):
   1. Core temperature > 38 °C or < 36 °C
   2. Heart rate > 90 / min
   3. Respiratory rate >20 / min, or PaCO2 < 32 mmHg, or mechanical ventilation
   4. White cell count > 12 x 10^9^ / L or < 4 x 10^9^ / L or > 10% immature neutrophils.
3. Treated with continuous vasopressors or inotropes to maintain a systolic blood pressure > 90 mmHg or mean arterial blood pressure > 60 mmHg or the target blood pressure set by the treating clinician for maintaining perfusion.
4. Administration of vasopressors or inotropes for ≥ 4 hours and present at time of randomization.

**Exclusion criteria:**

1. Age less than 18 years.
2. Met all inclusion criteria more than 24 hours before study inclusion.
3. Patient is pregnant and/or lactating.
4. Has been intubated (excluding time spent intubated within an operating theatre or transport) for greater than 12 hours in an intensive care unit before randomization.
5. Proven or suspected acute primary brain lesion such as traumatic brain injury, intracranial hemorrhage, stroke, or hypoxic brain injury.
6. Proven or suspected spinal cord injury or other pathology that may result in permanent or prolonged weakness.
7. Admission as a consequence of a suspected or proven drug overdose or burns.
8. Administration of ongoing neuromuscular blockade.
9. Mean arterial blood (MAP) pressure that is less than 50 mmHg despite adequate resuscitation and vasopressor therapy at time of randomization.
10. Heart rate less than 55 beats per minute unless the patient is being treated with a beta-blocker or a high grade atrio-ventricular block in the absence of a functioning pacemaker.
11. Known sensitivity to any of the study medications or the constituents of propofol (egg, soya or peanut protein).
12. Acute fulminant hepatic failure.
13. Patient has been receiving full time residential nursing care.
14. Treatment limitations: Death is deemed to be imminent or inevitable during this admission and either the attending physician, patient or substitute decision maker is not committed to active treatment.
15. Patient has an underlying disease that makes survival to 90 days unlikely.

**Table S1: Process of care**

|  | **DEX (n=44)** | **Usual care (n=39)** | **P value** |
| --- | --- | --- | --- |
| *Vasoactive drugs* |  |  |  |
| Number of days receiving a vasoactive agent | 3 [2, 5] | 3 [2, 7] | 0.35 |
| Patients with vasoactive drugs stopped < 48 hrs. after randomization – no. (%) | 21 (47.7) | 21 (53.8) | 0.58 |
| *Sedative and analgesic drugs* |  |  |  |
| Propofol – no. (%) | 42 (95.5) | 37 (94.9) | 0.90 |
| Total duration – hrs. | 28.6 [7.2, 104.0] | 63.7 [22.4, 186.0] | 0.03 |
| Cumulative daily dose (mg/kg/d) | 3.32 [1.09, 13.60] | 14.3 [6.11, 27.90] | <0.001 |
| Midazolam – no. (%) | 19 (43.2) | 29 (74.4) | 0.004 |
| Total duration – hrs. | 27.0 [5.9, 177.0] | 25.1 [4.1, 67.5] | 0.38 |
| Cumulative daily dose (mg/kg/d) | 0.09 [0.05, 0.19] | 0.10 [0.06, 0.22] | 0.64 |
| Fentanyl – no. (%) | 39 (88.6) | 37 (94.9) | 0.31 |
| Cumulative daily dose (mcg/kg/d) | 4.35 [2.30, 8.49] | 2.50 [1.69, 8.59] | 0.16 |
| Morphine – no. (%) | 17 (38.6) | 10 (25.6) | 0.21 |
| Cumulative daily dose (mg/kg/d) | 0.81 [0.22, 1.10] | 0.29 [0.12, 0.53] | 0.07 |
| Diazepam – no. (%) | 5 (11.4) | 0 (0) | 0.06 |
| Ketamine – no. (%) | 3 (6.8) | 1 (2.6) | 0.62 |
| Haloperidol – no. (%) | 7 (15.9) | 8 (20.5) | 0.59 |
| *Sedation level* |  |  |  |
| Percentage of RASS scores < -2 |  |  |  |
| Day 1 after randomization | 33.3 [16.7, 83.3] | 66.6 [33.3, 100] | 0.03 |
| Day 2 after randomization | 16.7 [0, 50] | 16.7 [0, 83.3] | 0.88 |
| Percentage of RASS scores between -2 and +1 |  |  |  |
| Day 1 after randomization | 41.7 [16.7, 66.7] | 20.0 [0, 66.7] | 0.05 |
| Day 2 after randomization | 83.3 [33.3, 100] | 83.3 [16.7, 100] | 0.98 |
| *24-hour fluid balance (ml)* |  |  |  |
| Day 1 after randomization | 490 [-315, 1767] | 303 [-670, 759] | 0.25 |
| Day 2 after randomization | -129 [-1440, 621] | -396 [-1246, 116] | 0.48 |
| *Daily urine output (ml)* |  |  |  |
| Day 1 after randomization | 1638 ± 1319 | 1212 ± 1077 | 0.11 |
| Day 2 after randomization | 1826 ± 1147 | 1496 ± 1136 | 0.23 |
| Number of days on RRT | 0 [0, 0.5] | 0 [0, 3] | 0.26 |

Categorical values are expressed as numbers (%). Continuous variables are presented as means ± SD if normally distributed, otherwise as medians [IQR]. RASS: Richmond Agitation-Sedation Scale. RRT: Renal replacement therapy.

**Table S2: Reported Adverse Events.**

| **Adverse events** | **DEX (n=44)** | **Usual care (n=39)** | **P value** |
| --- | --- | --- | --- |
| Days with delirium | 0 [0, 2] | 0 [0, 2.5] | 0.49 |
| Delirium at any point during stay | 13 (29.5) | 14 (35.9) | 0.54 |
| Adverse events | 9 (20.5) | 3 (7.7) | 0.10 |
| Bradycardia | 5 (11.4) | 0 (0) | 0.06 |
| Hypotension | 7 (15.9) | 1 (2.6) | 0.04 |
| Serious adverse events | 2 (4.5) | 1 (2.6) | 0.55 |

Values are expressed as numbers (%), or medians [IQR]. Table describes number of patients who experienced each event during the original SPICE III trial. Patients can have multiple AEs. Due to the un-blinded study design, events were reported by site investigators but not systematically collected in both groups. Bradycardia was defined as heart rate < 55 beats per minute requiring intervention e.g. pacing, pharmacological support, or modification of dexmedetomidine or other medication dose. Hypotension was defined as hypotension which is clinically significant in the Principal Investigator’s opinion.

**Table S3: Number (%) of patients recruited at each site.**

| **Site** | **All patients (n=83)** | **DEX (n=44)** | **Usual care (n=39)** |
| --- | --- | --- | --- |
| Austin Hospital, Melbourne | 39 (47.0) | 20 (45.5) | 19 (48.7) |
| University Hospital of Bern | 44 (53.0) | 24 (54.5) | 20 (51.3) |

Table S4: Clinical outcomes for DEX vs. Usual care in patients younger and older than the median age (63.7 years)

| **Outcome** | **Age < median (n=33)** | | **Age > median (n=50)** | |  |
| --- | --- | --- | --- | --- | --- |
|  | ***Median difference*** | ***P value*** | ***Median difference*** | ***P value*** | ***P value for interaction^c^*** |
| NEq dose*^a^* (µg/kg/min) | -0.01 [-0.08, 0.07] | 0.34 | -0.02 [0.08, 0.04] | 0.25 | 0.80 |
| Cumulative NEq dose*^a^* (µg/kg/48hrs.) | -0.35 [4.10, 3.40] | 0.32 | -0.86 [-3.80, 2.08] | 0.35 | 0.82 |
| Total duration of vasopressor support*^b^* (hrs.) | 5.9 [-63.8, 75.6] | 0.58 | 14.7 [-44.7, 74.2] | >0.99 | 0.81 |
| Duration of invasive ventilation*^b^* (days) | -2.2 [-5.8, 1.4] | 0.24 | 0.2 [-4.3, 4.6] | 0.97 | 0.43 |
| Hospital length of stay*^b^* (days) | -6.3 [-25.9, 13.3] | 0.17 | -0.6 [-7.9, 6.7] | 0.86 | 0.42 |
| ICU length of stay*^b^* (days) | -2.2 [-6.1, 1.8] | 0.27 | 0.2 [-5.8, 6.2] | 0.82 | 0.41 |
|  | ***Adjusted^d^ OR (95% CI)*** | ***P Value*** | ***Adjusted^d^ OR***  ***(95% CI)*** | ***P Value*** | ***P value for interaction^c^*** |
| Patients alive and vasopressor-free at 48 hrs. after randomization | 1.72 (0.33 - 9.09) | 0.51 | 0.56 (2.04 - 0.16) | 0.37 | 0.44 |
| ICU mortality | 0.28 (0.04 - 2.22) | 0.21 | 0.59 (0.10 - 3.57) | 0.56 | 0.52 |
| Hospital mortality | 0.28 (0.04 - 2.22) | 0.21 | 0.86 (0.20 - 3.70) | 0.84 | 0.37 |
| Day 90 mortality | 0.43 (0.07 - 2.70) | 0.36 | 1.04 (0.26 - 4.17) | 0.95 | 0.45 |

Values are expressed as numbers (%), or medians [IQR]. Abbreviations: DEX: Dexmedetomidine; ICU: Intensive Care Unit; NEq: Noradrenaline equivalents.

*^a^* in the first 48 hours after randomization. *^b^* within principal hospital admission, measured from randomization. ^c^ p-value for interaction between age group and treatment group. ^d^ Model adjusted for admission diagnosis, hospital site, baseline NEq/MAP ratio, continuous renal replacement therapy, age, administration of hydrocortisone and presence of liver cirrhosis. Odds ratio refers to the comparison between DEX and Usual care patients with DEX patients being used as the reference group.

Table S5: Comparison of sepsis severity and treatment of patients randomized before and after the publication of the 2016 Sepsis Guidelines (published in January 2017)

|  | **Before (n=63)** | **After (n=20)** | **P value** |
| --- | --- | --- | --- |
| *Vasoactive drugs* |  |  |  |
| Duration of vasopressors from randomization (hrs.) | 53.1 [19.6, 120.0] | 33.2 [15.8, 69.3] | 0.23 |
| Patients with vasoactive drugs stopped < 48 hrs. after randomization – no. (%) | 29 (46.0) | 13 (65.0) | 0.14 |
| NEq dose*^a^* (µg/kg/min) | 0.04 [0.02, 0.13] | 0.02 [0.01, 0.08] | 0.11 |
| Noradrenaline at randomization – no. (%) | 52 (83.9) | 19 (95.0) | 0.20 |
| Adrenaline at randomization – no. (%) | 5 (7.9) | 1 (5.0) | 0.66 |
| Vasopressin at randomization – no. (%) | 2 (3.2) | 1 (5.0) | 0.57 |
| Dobutamine at randomization – no. (%) | 4 (6.3) | 0 (0) | 0.57 |
| Hydrocortisone – no. (%) | 27 (42.9) | 8 (40.0) | 0.82 |
| *SOFA^b^ sub-scores at randomization* |  |  |  |
| Cardiovascular | 3 [3, 4] | 3 [3, 3.5] | 0.83 |
| Respiratory | 3 [2, 3] | 2 [2, 3] | 0.044 |
| Coagulation | 0 [0, 2] | 0 [0, 1] | 0.23 |
| Liver | 0 [0, 1.5] | 0 [0, 1] | 0.99 |
| Renal | 2 [0, 4] | 1 [0, 3] | 0.21 |
| Blood lactate level at randomization (mmol/L) | 1.8 [1.4, 2.8] | 2.1 [0.95, 3.0] | 0.46 |
| *24-hour fluid balance (ml)* |  |  |  |
| At randomization | 1110 [0, 2759] | 115 [-146, 1616] | 0.05 |
| Day 1 after randomization | 408 [-295, 1343] | -178 [-1475, 1653] | 0.27 |
| Day 2 after randomization | -279 [-1387, 460] | -356 [-1253, 271] | 0.98 |

Values are expressed as numbers (%), or medians [IQR]. NEq: Noradrenaline equivalents. SOFA: Sequential Organ Failure Assessment. *^a^* in the first 48 hours after randomization. *^b^* A cardiovascular SOFA score ≥ 2 and ongoing sedation were mandated by the inclusion criteria. Unsedated central nervous system SOFA score could not be reliably ascertained from the medical records.
