# Supplementary material for: The effect of dexmedetomidine on vasopressor requirements in patients with septic shock: a subgroup analysis of the Sedation Practice in Intensive Care Evaluation [SPICE III] Trial
Source: Crit Care. 2020 Jul 16;24:441. doi: 10.1186/s13054-020-03115-x (PMC7367420; doi:10.1186/s13054-020-03115-x)
Supplement: Supplementary file 2 — Additional file 2. [file 13054_2020_3115_MOESM2_ESM.docx]

**Additional file 2**

Supplement to: Cioccari L, Luethi N, Bailey M, et al. Hemodynamic effects of dexmedetomidine in patients with septic shock: a subgroup analysis of the sedation practice in intensive care evaluation [SPICE III] trial.

**SPICE III investigators**

SPICE III Management committee: Yahya Shehabi (Chair), Yaseen Arabi, Frances Bass, Rinaldo Bellomo, Simon Erickson, Belinda Howe (Senior Project Manager), Suhaini Kadiman, Colin McArthur, Lynnette Murray, Michael Reade, Ian Seppelt, Jukka Takala, Steve A Webb, Matthew P Wise.

SPICE III Writing committee: Yahya Shehabi, MD, PhD, Belinda Howe, RN, BAppScNur, MPH, Rinaldo Bellomo, MD, PhD, Yaseen M Arabi, MD, Michael J Bailey, PhD, MSc, BSc(Hons), Frances Bass, BN, GCCC, Suhaini Kadiman, MD, Colin McArthur MBChB, FANZCA, FCICM, Lynnette Murray BAppSci, FAIMS, Michael Reade, MBBS, MPH, PhD, Ian Seppelt, MBBS, BScMed, Jukka Takala, MD, PhD, Steve A Webb, MD, PhD, Matthew P Wise, MD, D.Phil.

SPICE III Affiliations of the writing committee: Australian and New Zealand Intensive Care Research Centre, Monash University, Melbourne, (MB, RB, BH, CM, LM, SAW); Monash University, School of Clinical Sciences, Melbourne (YS), Monash Health, Melbourne (YS); University of New South Wales, Clinical School of Medicine, Sydney (YS); King Saud Bin Abdulaziz University for Health Sciences and King Abdullah International Medical Research Center, Riyadh, Kingdom of Saudi Arabia (YA); King Abdulaziz Medical City, Riyadh, Kingdom of Saudi Arabia (YA); Royal North Shore Hospital, Sydney (FB); The George Institute for Global Health (FB); Austin Health, Melbourne (RB); National Heart Institute, Kuala Lumpur, Malaysia (SK); Auckland City Hospital, Auckland, New Zealand (CM); University of Queensland, Brisbane (MR); Royal Brisbane & Women’s Hospital, Brisbane (MR); Australian Defence Force, Brisbane (MR); Sydney Medical School – Nepean, University of Sydney, Sydney (IS); Dept of Clinical Medicine, Macquarie University, (IS); Department of Intensive Care Medicine, Inselspital, Bern University Hospital, University of Bern, Bern, Switzerland (JT); University of Bern, Bern, Switzerland (JT); St John of God Subiaco, Subiaco (SAW); University Hospital of Wales, Cardiff, United Kingdom (MPW); (all in Australia unless specified).

SPICE III Study coordinating centre: The Australian and New Zealand Intensive Care Research Centre (ANZIC-RC), School of Public Health and Preventive Medicine, Monash University, Melbourne. Michael J Bailey, Belinda D. Howe, Lynette Murray, Vanessa Singh.

Site investigators (alphabetically by institution and all in Australia unless specified as New Zealand [NZ], Ireland [IR], Italy [IT], Malaysia [MY], Saudi Arabia [SA],Switzerland [CH] or United Kingdom [UK]):Albury Base Hospital, Albury, NSW, C. Mashonganyika, H. McKee, A. Tonks; AltnagelvinArea Hospital, Londonderry, UK, A. Donnelly, N. Hemmings, S. O'Kane; Auckland CityHospital CVICU, Auckland, NZ, A. Blakemore, M. Butler, K. Cowdrey, J. Dalton, E. Gilder,S. Long, L. McCarthy, S. McGuinness, R. Parke; Auckland City Hospital DCCM, Auckland,NZ, Y. Chen, C. McArthur, R. McConnochie, L. Newby; Austin Health, Melbourne, VIC, R.Bellomo, G. Eastwood, L. Peck, H. Young; Bendigo Hospital, Bendigo, VIC, C. Boschert, J.Edington, J. Fletcher, J. Smith; Blacktown Hospital, Sydney, NSW, K. Nand, A. Raza, T.Sara; Bristol Royal Infirmary, Bristol, UK, J. Bennett-Britton, J. Bewley, V. Bodenham, L.Cole, K. Driver, L. Grimmer, L. Howie, C. Searles, K. Sweet, D. Webster; Central GippslandHealth, Sale, VIC, A. van Berkel, H. Connor, J. Dennett, M. van Der Graaff; ChristchurchHospital, Christchurch, NZ, S. Henderson, J. Mehrtens, K. Miller, E. Minto, A. Morris, S.Noble, K. Parker; Dandenong Hospital, Melbourne, VIC, L. Bulfin, N. Hart, K. Shepherd, S.Vij; Derriford Hospital, Derriford, UK, S. Dickson, E. Elloway, C. Ferguson, R. Jackson, P.MacNaughton, M. Marner, R. Squire, S. Waddy, P. Wafer, J. Welbourne; Dorset CountyHospital, Dorchester, UK, P. Ashcroft, D. Chambler, S. Dukes, A. Harris, S. Horton, S.Sharpe, P. Williams, S. Williams; Dunedin Hospital, Dunedin, NZ, M. Bailey, E. Blazquez,D. France, R. Hutchison, A. O'Connor; Gold Coast University Hospital, Gold Coast, QLD, G.Comadira, M. Gough, M. Tallott; Gosford Hospital, Gosford, NSW, M. Bastick, R. Cameron,S. Donovan, K. Ellis, A. Gaur, R. Gregory, J. Naumoff, E. Turner, M. White; Hornsby Ku-Ring-Gai Hospital, Sydney, NSW, KFJ. Au, J. Fratzia, S. Treloar; Hospital Pulau Pinang,Pulau Pinang, MY, CH. Lim, Maseeda.Y, AP. Tan, CL. Tang, CY. Yong; Inselspital BernUniversity Hospital, Bern, CH, M. Akaltan, S. Berger, D. Blaser, L. Fazlija, ML. Jong, M.Lensch, R. Ludwig, T. Merz, K. Nettelbeck, M. Roth, M. Schafer, J. Takala, A. Wehr, D.Zacharias; Institut Jantung Negara, Kuala Lumpur, MY, R. Amran, HN. Ashraf, N. Azmi, N.Basri, H. Burhanuddin, Y. Hadinata, A. Hamdan, S. Kadiman, AIYM. Rashid, IN. Sabran, S.Sulaiman, I. N. Zabidi; King Abdulaziz Medical City, King Saud Bin Abdulaziz Universityfor Health Sciences and King Abdullah International Medical Research Center, Riyadh, SA,A. Al-Dawood, M. Aljuaid, H. Al Anizi, A. AlSaeedi, Y. Arabi, M. Dbsawy, A. Deeb, M.Hegazy, M. Ibrahim, Kings College Hospital, London, UK, E. Clarey, E. Corcoran, C.Finney, C. Harris, P. Hopkins, H. Noble, J. Smith, L. Thompson, T. Williams; King SaudMedical City, Riyadh, KSA, LA. Dumlao, R. Bassam, MA. Hassan, N. Naseem, MH. Al Kurdi, AM. Al-Harthy; Knox Private Hospital, Melbourne, VIC, S. Bernard, L. Sebafundi, C.Serban; Kuala Lumpar General Hospital, Kuala Lumpur, MY, SK. Lim, N. Mazidah, N.Saidin, N. Sjamsuddin, ITA. Tan, N. Zabidi; Launceston General Hospital and Clifford CraigMedical Research Trust, Launceston, TAS, M. Brain, S. Mineall; Lyell McEwin Hospital,Adelaide, SA, M. Kanhere, N. Soar; Melaka General Hospital, Melaka, MY, N. Abd Kadir,NH. Abdullah, R. Awang, Z. Emperan, NS. Husin, NI. Ismail, SZ. Ismail, FNA. MohdKhadzali, MF. Norddin; Middlemore Hospital, Auckland, NZ, J. Aguila, C. Bold, B.Clatworthy, A. Dias, C. Hogan, A. Kazemi, V. Lai, R. Song, A. Williams; Monash MedicalCentre, Melbourne, VIC, D. Bhatia, L. Bulfin, S. Elliot, P. Galt, K. Lavrans, P. Ritchie, A.Wang; Nepean Hospital, Sydney, NSW, R. Gresham, J. Lowrey, K. Masters, P. Palejs, I.Seppelt, F. Symonds, L. Weisbrodt, C. Whitehead; Newcastle upon Tyne Hospitals (FreemanHospital and Royal Victoria Infirmary), UK, M. Babio-Galan, V. Calder, I. Clement, A.Harrison, I. McCullagh, C. Scott, L. Thompson; North Shore Hospital, Auckland, NZ, R.Bevan, S. Caniba, D. Hacking, L. Maher; IRCCS San Raffaele Scientific Institute, Milan, IT,ML. Azzolini, P. Beccaria, S. Colombo, G. Landoni, C. Leggieri, C. Luca, D. Mamo, E.Moizo, G. Monti, M. Mucci, A. Zangrillo; Prince of Wales, Sydney, NSW, M. Albania, S.Arora, Y. Shi; Prince Sultan Military Medical City, Riyadh, SA, A. Abudayah, G.Almekhlafi, E. Al Amodi, S. Al Samarrai, M. Badawi, R. Cubio Caba, O. Elffaki, Y.Mandourah, J. Valerio; Princess Alexandra Hospital, Brisbane, QLD, C. Joyce, J. Meyer, E.Saylor, B. Venkatesh, E. Venz, J. Walsham, K. Wetzig; Princess Royal University Hospital,London, UK, E. Clarey, C. Harris, P. Hopkins, H. Noble, L. Thompson, T. Williams; QueenElizabeth Hospital, MY, TM. Khoo, JES. Liew, AN. Sakthi, A. Zulkurnain; Queen ElizabethHospital Birmingham, Birmingham, UK, A. Bamford, C. Bergin, R. Carrera, L. Cooper, L.Despy, K. Ellis, S. Harkett, L. Mee, E. Reeves, C. Snelson, E. Spruce; Queen ElizabethHospital Kings Lynn, UK, G. Cooper, R. Hodgson, D. Pearson, M. Rosbergen; RajaPerempuan Zainab II Hospital, Kota Bharu, MY, MN. Ali, NI. Bahar, A. Ismail, WNW.Ismail, NM. Samat, NSM. Piah, R. Abd Rahman; Redcliffe Hospital, Brisbane, QLD, M.Duroux, M. Ratcliffe, T. Warhurst; Rotorua Hospital, Rotorua, NZ, U. Buehner, E. Williams;Royal Berkshire Hospital, Reading, UK, N. Jacques, L. Keating, S. Macgill, KL. Tamang, N.Tolan, A. Walden; Royal Bournemouth Hospital, Bournemouth, UK, R. Bower, J. Cranshaw,K. Molloy, S. Pitts; Royal Brisbane and Women’s Hospital, Brisbane, QLD, J. Butler, R.Dunlop, C. Fourie, P. Jarrett, M. Lassig-Smith, A. Livermore, S. O'Donoghue, M. Reade, T. Starr, J. Stuart; Royal Darwin Hospital, Darwin, NT, L. Campbell, M. Phillips, D. Stephens, J.Thomas; Royal Hobart Hospital, Hobart, TAS, D. Cooper, R. McAllister; Royal Infirmary of Edinburgh, Scotland, UK, G. Andrew, L. Barclay, H. Dawson, DM. Griffith, D. Hope, G.Wojcik, C. McCulloch, R. Paterson; Royal Liverpool Hospital, Liverpool, UK, L. Ascough,C. Paisley, J. Patrick-Heselton, D. Shaw, V. Waugh, K. Williams, I. Welters; RoyalMelbourne Hospital, Melbourne, VIC, D. Barge, A. Jordan, C. MacIsaac, T. Rechnitzer;Royal North Shore Hospital, Sydney, NSW, F. Bass, J. Gatward, N. Hammond, P. Janin, A.O'Connor, W. Stedman, E. Yarad; Sarawak General Hospital, Sarawak, MY, NA. Razak, N.Dzulkipli, SL. Jong, K. Asen, WL. Voon, S. Liew; St George's Hospital London, UK, J. Ball,V. Barnes, C. Dalton, S. Farnell-Ward, H. Farrah, K. Maher, J. Mellinghoff, C. Ryan, P.Shirley; St James University Hospital, Dublin, IR, L. Conlon, A. Glover, I. Martin-Loeches,E. O'Toole; St John of God Hospital Subiaco, Subiaco, WA, J. Ewan, J. Ferrier, E. Litton, SA.Webb; St Thomas Hospital, London, UK, , W. Berry, U. Blanco Alonso, A. Bociek, S.Campos, S. Jawara, F. Hanks, A. Kelly, K. Lei, C. McKenzie, M. Ostermann, R. Wan, StVincent's Hospital, Sydney, NSW, S. Al-Soufi, S. Leow, K. McCann, C. Reynolds; StVincent's University Hospital, Dublin, IR, K. Brickell, C. Fahey, L. Hays, N. Hyde, A.Nichol, D. Ryan; Sunshine Coast University Hospital and Nambour Hospital, Sunshine Coast,QLD, J. Brailsford, A. Buckley, L. Forbes, T. Maguire, J. Moore, L. Murray; The NorthernHospital, Melbourne, VIC, A. Ghosh, M. Park, S. Said; Toowoomba Hospital, Toowoomba,QLD, J. Smith, A. Visser; Universiti Sains Malaysia Hospital, MY, HZ. Abidin, S. Ali, MH.Hassan, SC. Omar, WFW. Shukeri; University College Hospital London, UK, D. Brealey, G.Bercades, E. Blackburn, N. Macallum, A. Macklin, JH. Ryu, K. Tam, D. Smyth; UniversityHospital of Coventry and Warwick, Coventry, UK, A. Arif, C. Bassford, C. Morgan, C.Swann, G. Ward, L. Wild; University Hospital Geelong, Geelong, VIC, A. Bone, T. Elderkin,D. Green, D. Sach, T. Salerno, N. Simpson; University Hospital of North Tees, Stockton-on-Tees, UK, F. Brohi, M. Clark, L. Williams; University Hospital of Wales, Cardiff, UK, J.Brooks, E. Cocks, J. Cole, J. Curtin, R. Davies, H. Hill, M. Morgan, N. Palmer, C. Whitton,M. Wise; University Malaya Medical Center, MY, P. Baskaran, MS. Hasan, LY. Tham;Wellington Regional Hospital, Wellington, NZ, R. Sol Cruz, D. Dinsdale, S. Edney, C. Firkin,F. FitzJohn, G. Hill, A. Hunt, S. Hurford, G. Jones, H. Judd, C. Latimer-Bell, C. Lawrence, E. Lesona, L. Navarra, Y. Robertson, H. Smellie, AM. Vucago, P. Young; Western GeneralHospital Scotland, Edinburgh, UK, H. Dawson, DM. Griffith, R. Paterson; WestmeadHospital, Sydney, NSW, P. Clark, J. Kong, J. Ho, V. Nayyar, C. Skelly.
